# Draft Genome Sequence of the Halophilic Bacterium *Halobacillus* sp. Strain BAB-2008

**DOI:** 10.1128/genomeA.00222-12

**Published:** 2013-02-21

**Authors:** M. N. Joshi, A. S. Pandit, A. Sharma, R. V. Pandya, A. K. Saxena, S. B. Bagatharia

**Affiliations:** Gujarat State Biotechnology Mission, Department of Science & Technology, Gujarat, India

## Abstract

The *Halobacillus* sp. strain BAB-2008 is a moderately halophilic, rod-shaped, Gram-positive, orange-pigmented, carotenoid-producing bacterium isolated from saline soil near Zazam-Solar Park Road, Gujarat, India. Here we present the 3.7-Mb genome sequence to provide insights into its functional genomics and potential applications for carotenoid and enzyme production.

## GENOME ANNOUNCEMENT

The genus *Halobacillus* was identified by Spring et al. (1) and comprises 18 species with validly published names: *H. halophilus*, *H. litoralis*, and *H. trueperi* (1), *H. salinus* (2), *H. karajensis* (3), *H. locisalis* (4), *H. aidingensis* and *H. dabanensis* (5), *H. yeomjeoni* (6), *H. campisalis* (7), *H. profundi* and *H. kuroshimensis* (8), *H. faecis* (9), *H. mangrovei* (10), *H. alkaliphilus* (11), *H. naozhouensis* (12), *H. salsuginis* (13), and *H. seohaensis* (14). The *Halobacillus* genus comprises moderate halophiles which can grow and produce enzymes over a very wide range of salinities, making them very attractive for research and for screening of novel enzymes with unusual properties (15). Many moderate halophiles produce carotenoids as a protective mechanism against photooxidation processes. Carotenoids have major applications in the food industry as food-coloring agents and as additives in health food products (16). The halophilic aspect of these bacteria has exciting potential, for instance, in their possible application in agriculture to construct salt-resistant plants carrying prokaryotic genes encoding enzymes for the synthesis of osmoprotective compounds (17).

The halophilic bacterium *Halobacillus* sp. strain BAB-2008 was isolated from a soil sample near Zazam-Solar Park Road, District Patan (23°55′782″N, 71°18′480″E), Gujarat, India, by using the traditional dilution-plating method. Preliminary characterization revealed that *Halobacillus* sp. BAB-2008 is Gram positive, orange pigmented, and halophilic (can grow at up to 15% NaCl).

Whole-genome sequencing of the strain was done with a high-throughput Ion Torrent Personal Genome Machine with an Ion Torrent Server (Torrent suite version 3.2). Following the manufacturer’s protocol, 22.11× coverage data and a total of 1,078,490 mate-paired reads (shortest read with 91 bp and longest read with 176 bp) were obtained. *De novo* assembly was performed using the MIRA-3 assembler (version 3.1.0). The automatic annotation of the genome was performed using the NCBI Prokaryotic Genomes Automatic Annotation Pipeline (PGAAP) (http://www.ncbi.nlm.nih.gov/genomes/static/Pipeline.html), which utilizes GeneMark (18), Glimmer (19), and tRNAscan-SE searches (20), and using the RAST server (21) with the SEED database (22). Functional annotation of genes was performed using the KEGG Automatic Annotation Server (23).

The total length of the genome was found to be 3,784,751 bp, which was distributed in 137 contigs (scaffold *N*_50_ = 51,319 bp). The G+C content was 46.83%. The *Halobacillus* sp. BAB-2008 harbored 410 subsystems, having 3,935 protein-coding genes, 60 tRNAs, and 9 rRNAs. Carotenoid biosynthesis genes (*crtN*, *crtP*, *crtB*, and *crtM*) present on the chromosome of *Halobacillus* sp. BAB-2008 indicate that this species might have possible applications in carotenoid production. Genes encoding serine proteinases, amylases, nucleases, esterases, and lipases were also identified on the chromosome.

Thus, the ability to grow under halophilic conditions, the presence of a clearly defined carotenoid biosynthetic pathway, and the probable potential toward enzyme production make *Halobacillus* sp. BAB-2008 an organism of industrial importance. Moreover, this bacterium may be used as a model organism for molecular study of the osmoregulatory mechanisms which help moderate halophiles grow over a wide range of salt concentrations.

### Nucleotide sequence accession number. 

The draft genome sequence of *Halobacillus* sp. BAB-2008 has been deposited at GenBank under the accession number ANPF00000000.
